# Biotransformation of the Fluorinated Nonsteroidal Anti‐Inflammatory Pharmaceutical Flurbiprofen in Activated Sludge Results in Accumulation of a Recalcitrant Fluorinated Aromatic Metabolite

**DOI:** 10.1002/gch2.201800093

**Published:** 2019-01-16

**Authors:** Kadir Yanaç, Robert W. Murdoch

**Affiliations:** ^1^ Department of Environmental Engineering Middle East Technical University Üniversiteler Mahallesi Dumlupınar Bulvarı No: 1 06800 Ankara Turkey; ^2^ Biotechnology Department Graduate School of Natural and Applied Sciences Middle East Technical University Inonu Bulvarı 06531 Ankara Turkey

**Keywords:** biodegradation, flurbiprofen, nonsteroidal anti‐inflammatories, NSAID, pharmaceuticals

## Abstract

Flurbiprofen is a fluorinated, nonsteroidal, anti‐inflammatory pharmaceutical with potential application in a wide range of maladies. Currently, there is no information regarding its environmental fate. To address this, flurbiprofen is spiked at 500 and 50 ppm into activated sewage sludge taken from the municipal treatment plant of Ankara, Turkey. Flurbiprofen is partially degraded after 80 days, with removal proportion varying from 33% to 48%. Isolation of organisms able to use flurbiprofen as a sole carbon and energy source is unsuccessful. A transient, acid‐labile yellow coloration appears in supernatants after addition of flurbiprofen. During disappearance, a novel potential metabolite is detected by high‐performance liquid chromatography (HPLC) analyses, a chemical that does not appear in killed controls or in nonflurbiprofen‐amended controls. Mass spectra of the novel chemical obtained at low and high collision energies are consistent with 4‐(1‐carboxyethyl)‐2‐fluorobenzoic acid, suggesting the application of a canonical metabolic paradigm for halogenated biphenyl metabolism by bacteria in which the nonhalogenated ring is metabolized by dioxygenation and metacleavage, leaving the halogenated aromatic ring behind. This metabolite shows no signs of disappearance after the 80‐day monitoring period, implying that the environmental release of flurbiprofen might be of concern.

## 1 Introduction

Flurbiprofen (2‐(3‐fluoro‐4‐phenyl‐phenyl)propanoic acid) is a type of nonsteroidal anti‐inflammatory (NSAID) pharmaceutical. NSAIDs are one of the most consumed drug classes across the world and have been shown to have potential environmental health impacts.^1^

Global sales of flurbiprofen have been estimated at $100 million annually with approximately 5% annual growth.^2^ While in the United States, flurbiprofen is a prescription‐only medication that sees limited usage, in Turkey, a top‐20 global economy, flurbiprofen is the most popular NSAID and the third most popular pharmaceutical overall.^2^ Additionally, new investigations suggest that flurbiprofen may be used for treatment of prostate and colon cancers^3^ and for anti‐obesity purposes,^4^ implying that its popularity might surge in coming decades.

Unlike most popular NSAIDs, flurbiprofen is fluorinated. Fluorine is generally incorporated into pharmaceuticals in order to increase their biological half‐lives. On the other hand, introduction of fluorine into chemicals creates environmental problems due to increased lipophilicity and recalcitrance.^5^ Currently, approximately 25% of pharmaceuticals are fluorinated, a percentile that will likely continue to increase.^6^, ^7^

Approximately 22% of consumed flurbiprofen is excreted unmodified or as an easily hydrolysable phase II metabolic conjugate.^8^, ^9^ Few studies have examined flurbiprofen environmental concentrations. 0.21 and 0.34 µg L^−1^ flurbiprofen were detected in WWTP effluents of France and Italy, respectively.^10^ No flurbiprofen was detected in Swedish WWTP effluents.^11^ There are no reports related to fate, ecotoxicity or removal of flurbiprofen, likely because it is not popular in countries where scientific research is dense. Flurbiprofen probably has high sorption ability and bioaccumulation potential in the environment given its poor water solubility and *K*_ow_ of 4.2.^12^

There is currently no ecotoxicological information regarding flurbiprofen. In Europe, two other common NSAIDs, diclofenac and ibuprofen, were recently considered for inclusion in a list of priority water contaminants considering the results of ecotoxicity studies.^13^ Environmentally relevant concentrations of these NSAIDs and their metabolites have been demonstrated to have toxic effects against a wide variety of aquatic organisms such as mussels,^14^, ^15^, ^16^, ^17^, ^18^ insects,^19^ and fish.^20^ Additionally, pharmaceuticals occur in the environment as complex mixtures rather than isolated chemicals, which may produce greater toxic effects on living organisms.^21^, ^22^, ^23^

**Figure 1:**
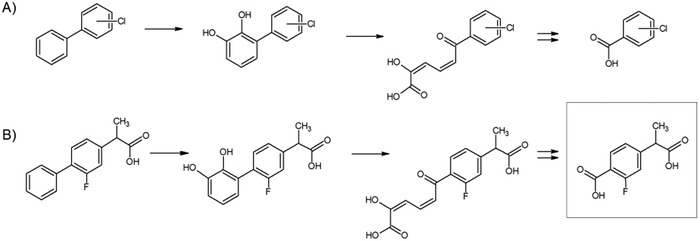
A) Monochlorinated biphenyl pathway.^26^ B) Proposed pathway for FLB degradation; the final metabolite, 4‐(1‐carboxyethyl)‐2‐fluorobenzoic acid (indicated by the surrounding box), was detected by LCMS in this study.

While there have been reports of fungal and bacterial transformations of flurbiprofen in pure culture systems, there have yet to be any published reports of in situ fate, assimilative metabolism, or isolation of biodegrading bacteria.^24^ To date, reports of biodegradation of other bicyclic NSAIDS such as ketoprofen and mefenamic acid are similarly scarce.^25^ Application of one of the standard aromatic degradation paradigms, that for monochlorbiphenyl by which the unsubstituted ring is attacked by dioxygenative ring cleavage,^26^ predicts the accumulation of a 3‐fluorophenylacetic acid in the cell (**Figure** **1**).

Information regarding fluorinated biphenyls by bacteria is rarer. *Pseudomonas pseudoalcaigenes* KF707 degrades 2‐ and 4‐fluorobiphenyl via a standard biphenyl degradation pathway^27^ that yields 2‐ and 4‐fluorobenzoate as end products. In the case of fluorine substitution not confined to the one ring, both KF707 and *Burkholderia* sp. LB400 degraded 4,4′‐difluorobiphenyl.^28^ It was also demonstrated that 2,2′‐difluorobiphenyl was transformed to 2′‐fluoro‐2,3‐dihydroxybiphenyl via BphA by *Burkholderia* sp. LB400.^29^

The purpose of this research is to examine the potential for and strategies employed in the biodegradation of flurbiprofen by environmental bacteria. This was approached by attempting to isolate organisms able to utilize flurbiprofen as sole carbon and energy source and by directly spiking sewage sludge with flurbiprofen and monitoring its fate.

## 2 Results

### 2.1 Monitoring Flurbiprofen Disappearance and Attempts to Enrich and Isolate

While initial enrichment for and isolation of mTAA and pTAA degrading bacteria were successful, multiple attempts to enrich for FLB degraders proved unsuccessful. This was in spite of several different batches of sludge from Ankara and from other cities, use of lower concentrations of FLB, application of a different mineral salt medium, addition of yeast extract, and use of different water sources.

Following addition of FLB to aerobic sewage sludge samples, a faint yellow coloration with a broad absorption peak ≈390 nm appeared after several days. This did not occur in nonamended controls. This yellow color was located in the supernatant following centrifugation and was acid labile, disappearing with acidification and reappearing upon neutralization.

**Figure 2:**
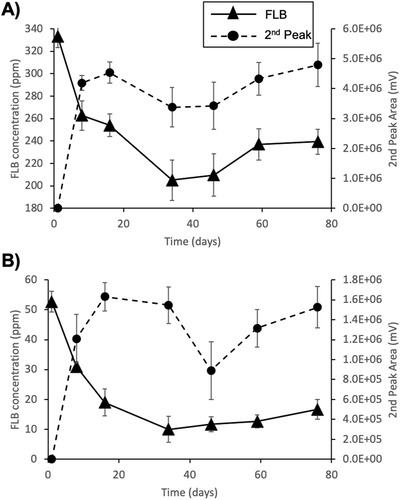
Time‐course of flurbiprofen concentration and presumptive metabolite appearance (following addition of A) 50 ppm FLB and B) 500 ppm FLB. Error bars represent standard deviation. Each experiment was conducted in triplicate.

Monitoring of FLB concentration over a span of 80 d revealed that most degradation took place over the first 34 d in the samples spiked with 500 ppm and with 50 ppm (**Figure** **2**). No notable changes in FLB concentration were detected in abiotic controls (data not shown). In either case, disappearance was not complete by the end of the 80 d sampling period, with 32 ± 6.3% and 47.8 ± 2.2% remaining in the 50 ppm and 500 ppm FLB amended samples, respectively.

**Figure 3:**
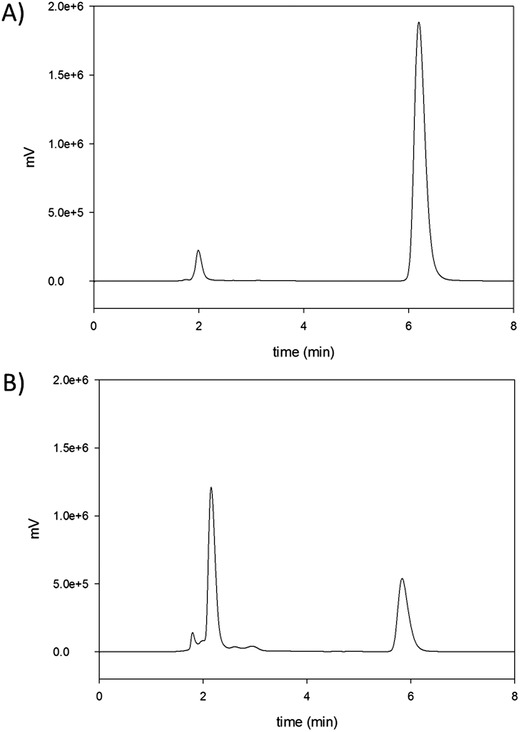
HPLC chromatograms following addition of 500 ppm FLB to sludge sample at A) day 0 and at B) day 8. Detection was at 247 nm. FLB eluted at ≈6 min while a second novel peak can be seen in b) at ≈2.2 min.

During degradation, a second peak eluting faster from the high performance liquid chromatography (HPLC) column and also absorbing at 247 nm was noted (**Figure** **3**B).

This second peak was fractionated and a superior HPLC protocol was developed for better separation and detection. In non‐FLB‐spiked sludge control samples, no FLB or second peak were detected, indicating that FLB is not present in ATMWTP sludges at ppm levels and that the second peak was dependent upon addition of FLB. Considering all time points in both sets of FLB‐spiked samples, the area of the second peak was proportional to the amount of FLB disappearance (*R* ^2^ = 0.78). This proportionality to the disappearance of FLB, the time‐course of its appearance, and nonpresence in abiotic and non‐FLB‐spiked controls indicate that the second peak represented an FLB biodegradation product.

### 2.2 Characterization of Flurbiprofen Metabolite by Liquid Chromatography Mass Spectroscopy (LCMS)

**Figure 4:**
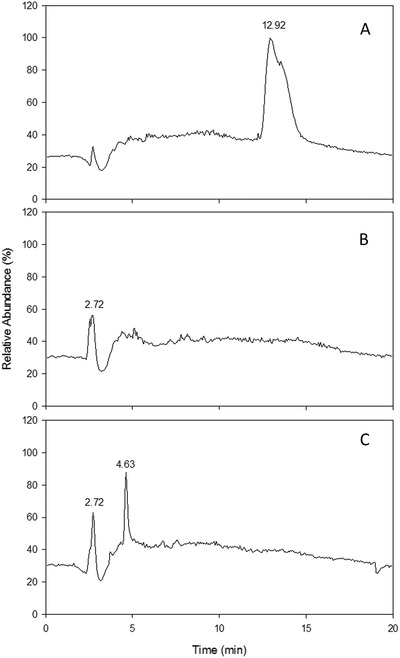
LCMS chromatograms of A) 500 ppm FLB in methanol, B) sludge blank sample, and C) sludge sample spiked with FLB. The mass spectra of the novel peak at 4.63 min in part (C) were examined closely for structural characterization.

The solvent gradient separation applied in LCMS resulted in clean separation of the presumptive metabolite at 4.63 min, a peak that did not appear in nonspiked sludge nor in the FLB standard (**Figure** **4**).

**Figure 5:**
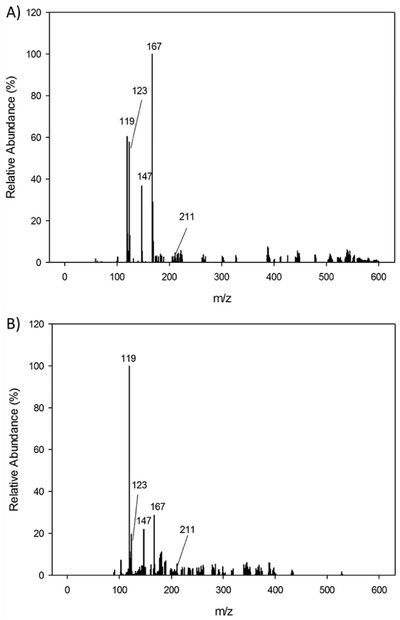
TOF MS ES‐spectrum of 4.6 min peak (putative FLB metabolite) at A) low collision energy (6 V) and B) high collision energy (15 V).

The mass spectrum of FLB was in accordance with known standards, with the parent ion at 243 m/z and major ion at 199 m/z. The second peak was subjected to two collision energies, 15 and 6 V under negative ionization. The resulting mass spectra were not consistent with any reference database compounds. At low collision energy, a major ion of 167 m/z was observed (**Figure** **5**A). At high collision energy, more fragmentation was observed, as expected, with the major ion at 119 m/z (Figure 5B).

**Figure 6:**
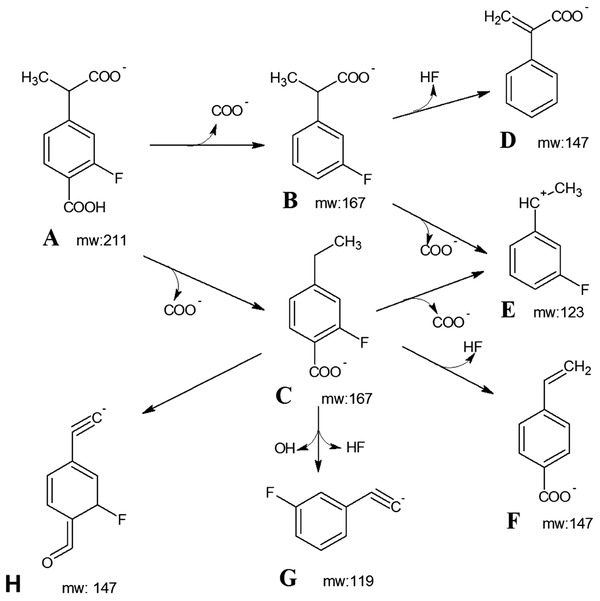
Prediction of MS fragmentation of 4‐(1‐carboxyethyl)‐2‐fluorobenzoic acid, based on CFM‐ID computational prediction algorithm.^30^

Referencing a known pathway for monochlorinated biphenyl,^26^ in which the nonhalogenated ring is subjected to dioxygenation, meta‐cleavage, and stepwise decarboxylation, a predicted metabolite was identified; 4‐(1‐carboxyethyl)‐2‐fluorobenzoic acid, a compound which when negatively ionized yields a molecular weight of 211 (**Figure** **6**, molecule A).

Fragmentation of 4‐(1‐carboxyethyl)‐2‐fluorobenzoic acid (a previously uncharacterized chemical) was predicted using a guide for mass spectral prediction^31^ and the CFM‐ID program, which models MS fragmentation^30^ (Figure 6). While the presumed parent ion of 211 m/z was detected, it failed to rise above the background noise. However, the key peaks at 119, 123, 147, and 167 m/z were fully consistent with the ions predicted by both MS fragmentation prediction approaches.

## 3 Discussion

While monocyclic NSAIDs such as ibuprofen have been widely reported to be readily mineralized,^32^, ^33^ the halogenated bicyclic NSAID diclofenac has become a concern due to its recalcitrance.^25^ Fluorination of organics including pharmaceuticals has become widespread, possibly as strategy to avoid the negative perception of chlorinated organics while still imparting desirable properties such as hydrophobicity and recalcitrance. In the increasingly popular fluorination of pharmaceuticals, a fluorine or trifluoro‐methyl group is used in place of a hydrogen atom or hydroxyl group; often the moiety targeted for replacement is the target of human phase I metabolic activity;^8^, ^9^ in other words, specifically to inhibit biotransformation.

Despite multiple sludge sources and techniques, attempts to enrich for and isolate bacteria capable of using flurbiprofen as sole carbon and energy source were not successful. Disappearance of FLB did not occur in abiotic (autoclaved) controls, indicating that some biotransformation was taking place. During disappearance, a faint, transient, acid‐labile yellow color appeared, indicating the presence of a meta‐cleavage product typical of bacterial aromatic dioxygenation pathways. A second chemical accumulated during biodegradation in a time‐ and FLB concentration‐dependent manner. This chemical did not appear in non‐FLB‐amended samples nor in abiotic controls, suggesting that it was a FLB metabolite. LCMS spectra of this metabolite were consistent with 4‐(1‐carboxyethyl)‐2‐fluorobenzoic acid, a mono‐fluorinated aromatic acid. This metabolite is in turn consistent with the application of the canonical chlorinated biphenyl pathway, in which the non‐halogenated ring is dioxygenated and subjected to meta‐ring‐cleavage^29^ (Figure 1).

It appears that in aerobic sludge, FLB is subjected to a single initial ring cleavage, after which a fluorinated metabolite accumulates (Figure 1). There was no indication that the metabolite, 4‐(1‐carboxyethyl)‐2‐fluorobenzoic acid, disappeared during the 80 days of monitoring the samples.

**Figure 7:**
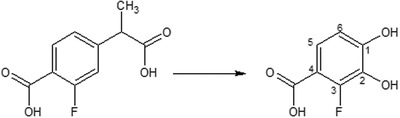
Potentially cytotoxic 3‐fluoro‐4‐carboxycatechol (right) which could be produced by action of the *ipf* pathway on the presumptive FLB metabolite (left).

Successful degradation of 2‐ and 4‐fluorobenzoates has been reported many times, while 3‐fluorobenzoates seem to be less often degraded efficiently due to the production toxic intermediates.^34^ 3‐Fluorocatechol is strongly resistant against ring‐cleavage enzymes, has tendency to accumulate, and has specific and severe toxic effects.^35^, ^36^, ^37^ If we apply the only described mechanism for aerobic substituted phenylacetic acid strategies, the *ipf* system, whereby the acetic acid group would be removed by an initial 1,2 dioxygenation,^32^, ^38^ we would see the production of a 3‐fluoro‐4‐phenylcatechol (**Figure** **7**). This may prove to be cytotoxic in a manner related to that seen for 3‐fluorocatechol, which is traditionally used to irreversibly poison ring cleavage systems.^34^, ^35^, ^36^, ^37^, ^39^ Thus, it is possible that metabolism of flurbiprofen leads to production of a cytotoxic fluorinated catechol, which specifically deactivates ring‐cleavage enzymes among other toxic effects. This could explain the poor rate of FLB degradation, the accumulation of 4‐(1‐carboxyethyl)‐2‐fluorobenzoic acid, and the failure to isolate FLB‐degrading organisms.

Altogether, it appears that flurbiprofen biodegradation is slow, incomplete, and can result in a remarkably recalcitrant dead‐end metabolite with a mass spectrum consistent with 4‐(1‐carboxyethyl)‐2‐fluorobenzoic acid, a chemical which has been previously undetected and is entirely uncharacterized. Of note, it is important to investigate whether lower, environmentally‐relevant conditions, FLB is likewise incompletely metabolized and whether the same metabolite accumulates.

## 4 Experimental Section

*Sludge* *Collection and Chemical Amendment*: Aerobic sewage sludge from the discharge line of the secondary sedimentation tank of Tatlar Municipal Wastewater. Treatment Plant in Ankara, Turkey (ATMWTP) was the primary target for enrichment and for spiking experiments. Mixed liquor suspended solids (MLSS) and mixed liquor volatile suspended solids (MLVSS) of aerobic sludge from ATMWTP were reported to range between 2.3 and 3.3 g L^−1^ and 1.66–2.73 g L^−1^, respectively, and soluble COD of 43 mg L^−1^.^40^ Isolation was also attempted from aerobic sludge from Yozgat and Eskişehir Municipal Wastewater Treatment Plants (Turkey). For all experiments, sludge was thoroughly resuspended and homogenized by shaking prior to aliquoting to sample flasks. For addition of flurbiprofen (FLB), m‐tolylacetic acid (mTAA), and p‐tolylacetic acid (pTAA), chemicals were dissolved in 1 mL of methanol prior to addition to the sludge aliquot.

*Enrichment and Isolation*: Enrichment and isolation of bacteria able to use flurbiprofen as sole carbon and energy source were performed via standard procedures: 500 ppm of substrate was spiked into 250 mL sewage sludge and shaken at 120 rpm at 30 °C. Substrate concentration was monitored by HPLC. In addition to flurbiprofen (FLB), enrichments were performed on p‐tolylacetic acid (pTAA) and m‐tolylacetic acid (mTAA) for the sake of positive controls for enrichment and isolation. When disappearance of substrate exceeded 50%, 100 µL of the enrichment was spiked into mineral salts medium (MSM)^41^ containing 500 ppm of the appropriate substrate. This serial enrichment was repeated two more times, for a total of three MSM transfers. Colonies were isolated from the final transfer by streaking onto MSM agarose plates with 250 ppm substrate. As initial isolations for organisms able to use FLB as sole carbon and energy source were not successful, several alternative methods were attempted, including lower FLB concentration throughout (50 and 250 ppm), supplementation with 2 ppm yeast extract, a different minimal medium recipe (M9 mineral medium)^42^ and alternative sources of water (sterilized tap water and commercial spring water rather than ddH_2_O).

*Flurbiprofen Disappearance and Transformation*: Flurbiprofen disappearance was monitored by spiking 50 and 500 ppm FLB into aerobic sludge samples from ATMWTP and shaken at 120 rpm at 30 °C. FLB was measured by HPLC over the course of 80 d. This experiment was conducted in triplicate. Two controls were applied; a non‐FLB‐spiked control and an abiotic control in which the sludge was autoclaved at 121 °C for 1 h prior to FLB addition.

*Sample Preparation*: Prior to HPLC analyses, sludge samples were centrifuged and the supernatants were filtered with syringe filters (nonsterile, 15 mm diameter, hydrophilic, 0.2 µm pore size, regenerated cellulose membrane, with polypropylene housing, Minisart, Germany). To account for substrate‐bound analytes, an extraction method was applied which entailed; lyophilization at −55 °C, extraction with acetone with 10 min of vortexing and 40 min sonication followed by centrifugation, removal, and filtration of the supernatant. Total substrate concentration is given as the sum of aqueous and solid phase concentrations. This procedure resulted in 67% recovery of spiked FLB across the working concentration.

*HPLC*: The concentrations of FLB, mTAA, and pTAA were monitored by HPLC. The HPLC device consisted of a system controller (Shimadzu, SCL‐10A VP, Kyoto, Japan) connected to a PC, a pump (SHIMADZU, LC‐10AT VP, Kyoto, Japan), a low‐pressure gradient unit (SHIMADZU, FCV‐10AL VP, Kyoto, Japan), a degasser (Shimadzu, DGU‐14A, Kyoto, Japan), a UV–vis detector (Shimadzu, SPD‐10A VP, Kyoto, Japan), a column oven (Shimadzu, CTO‐10A VP, Kyoto, Japan) and a reverse‐phase column (Macherey‐Nagel, CC 250/4 Nucleosil 50–5C 18ec., Düren, Germany).

An appropriate HPLC method was developed for separation and quantification of each chemical and for detection and fractionation of a second peak which appeared during FLB degradation in aerobic sludge in flasks (Table S1, Supporting Information).

Spiked chemicals were quantified by application of standard curves. A second peak representing a putative FLB metabolite was initially observed using the solvent and detection wavelength for FLB, although for subsequent quantification assays, a more appropriate method was developed and applied (Table S1, Supporting Information).

*Characterization of FLB Degradation by LCMS*: LCMS analyses of a blank sample, a 500 ppm FLB standard sample and an aerobic sludge sample spiked with 500 ppm FLB and taken after ≈50% FLB degradation (as determined by monitoring with HPLC) were carried out using a Waters (Milford, MA, USA) Acquity UPLC connected to Waters Synapt G1 MS (Milford, MA, USA) mass spectrometer in negative mode. The liquid chromatography column was an Acquity UPLC BEH C18 (Milford, MA, USA) 1.7 µm 1.0 × 100 mm operated at 35 °C with a solvent flow rate of 0. 03 mL min^−1^. A solvent gradient was applied, consisting of 30:70 methanol:40 × 10^−3^ m acetic acid (MeOH:AcAcid) for 15 min, followed by 60:40 MeOH:AcAcid for min 15–19 and 100% MeOH for 1 min.

The mass spectrometer was operated in ESI‐mode with 3 kV capillary voltage with 80 °C source temperature and 350 °C desolvation temperature. Two analyses were performed, high collision energy (15 V) and low collision energy (6 V). Detection was performed over a mass interval of 50 to 600 Da.

*Free Fluoride Detection*: A spectrophotometric technique was applied in order to investigate the release of fluoride ions during degradation of FLB.^43^ 0.25 mL cerous nitrate, 0.25 mL alizarine complexone, and 0.5 mL of sample were directly mixed and allowed to stand for 1 h at room temperature. In case of existence of fluoride, the mix gives a blue or light lilac color with a maximum absorbance of 625 nm. The method was applied to a standard curve of fluoride ions and was found to perform well over a range of 1 to 20 mg L^−1^ (*R* ^2^ = 0.98).

## Conflict of Interest

The authors declare no conflict of interest.

## Supporting information

Supplementary
