# Supplementary material for: Biotransformation of the Fluorinated Nonsteroidal Anti‐Inflammatory Pharmaceutical Flurbiprofen in Activated Sludge Results in Accumulation of a Recalcitrant Fluorinated Aromatic Metabolite
Source: Glob Chall. 2019 Jan 16;3(6):1800093. doi: 10.1002/gch2.201800093 (PMC6551406; doi:10.1002/gch2.201800093)
Supplement: Supplementary file 1 — Supplementary [file GCH2-3-1800093-s001.pdf]

Copyright WILEY-VCH Verlag GmbH & Co. KGaA, 69469 Weinheim, Germany, 2019.

Global Challenges
Open Access

Supporting Information

for *Global Challenges,* DOI: 10.1002/gch2.201800093

Biotransformation of the Fluorinated Nonsteroidal Anti-Inflammatory Pharmaceutical Flurbiprofen in Activated Sludge Results in Accumulation of a Recalcitrant Fluorinated Aromatic Metabolite

*Kadir Yanaç and Robert W. Murdoch**

Table S1. HPLC methods for FLB, mTAA, pTAA, and 2nd peak.

Chemical	Solution		Detection Wavelength (nm)	Oven Temperature (°C)	Elution Time (min)
	40 mM Acetic acid in water (%)	Methanol (%)			
FLB	40	60	247	60	6.0
mTAA	60	40	212	60	5.2
pTAA	60	40	212	60	5.2
2nd Peak	70	30	210	60	4.8
